# Soluble ST2 is a Useful Biomarker for Grading Cerebral–Cardiac Syndrome in Patients after Acute Ischemic Stroke

**DOI:** 10.3390/jcm9020489

**Published:** 2020-02-11

**Authors:** Pei-Hsun Sung, Hung Sheng Lin, Kuan-Hung Chen, John Y. Chiang, Sheung-Fat Ko, Pei-Lin Shao, Hsin-Ju Chiang, Chi-Hsiang Chu, Yi-Chen Li, Han-Tan Chai, Kun-Chen Lin, Hon-Kan Yip

**Affiliations:** 1 Division of Cardiology, Department of Internal Medicine, Kaohsiung Chang Gung Memorial Hospital and Chang Gung University, College of Medicine, Kaohsiung 83301, Taiwan; e12281@cgmh.org.tw (P.-H.S.); loveweib@gmail.com (C.-H.C.); ryichenli@gmail.com (Y.-C.L.); chaiht@mail.cgmh.org.tw (H.-T.C.); 2 Center for Shockwave Medicine and Tissue Engineering, Kaohsiung Chang Gung Memorial Hospital, Kaohsiung 83301, Taiwan; 3 Department of Neurology, Kaohsiung Chang Gung Memorial Hospital and Chang Gung University College of Medicine, Kaohsiung 83301, Taiwan.; a2522kimo@yahoo.com.tw; 4 Department of Anesthesiology, Kaohsiung Chang Gung Memorial Hospital and Chang Gung University, College of Medicine, Kaohsiung 83301, Taiwan; amigofx35@gmail.com; 5 Department of Computer Science & Engineering, National Sun Yat-sen University, Kaohsiung 80424, Taiwan; chiang@mail.cse.nsysu.edu.tw; 6 Department of Healthcare Administration and Medical Informatics, Kaohsiung Medical University, Kaohsiung 80708, Taiwan; 7 Department Radiology, Kaohsiung Chang Gung Memorial Hospital and Chang Gung University, College of Medicine, Kaohsiung 83301, Taiwan; sfatko@adm.cgmh.org.tw; 8 Department of Nursing, Asia University, Taichung 41354, Taiwan; m8951016@gmail.com; 9 Department of Obstetrics and Gynecology, Kaohsiung Chang Gung Memorial Hospital and Chang Gung University College of Medicine, Kaohsiung 83301, Taiwan; n22370@cgmh.org.tw; 10 Chung Shan Medical University School of Medicine, Taichung 40201, Taiwan; 11 Institute for Translational Research in Biomedicine, Kaohsiung Chang Gung Memorial Hospital and Chang Gung University, College of Medicine, Kaohsiung 83301, Taiwan; 12 Department of Medical Research, China Medical University Hospital, China Medical University, Taichung 40402, Taiwan

**Keywords:** soluble ST2, inflammatory biomarkers, ischemic stroke, left ventricular function, cerebral–cardiac syndrome

## Abstract

This study tested whether the soluble (s)ST2 is a superb biomarker predictive of moderate to severe cerebral–cardiac syndrome (CCS) (defined as coexisting National Institute of Health Stroke Scale (NIHSS) >8 and left-ventricular ejection fraction (LVEF) <60%) in patients after acute ischemic stroke (IS). Between November 2015 and October 2017, a total of 99 IS patients were prospectively enrolled and categorized into three groups based on NIHSS, i.e., group 1 (NIHSS ≤ 8, *n* = 66), group 2 (NIHSS = 9-15, *n* = 14) and group 3 (NIHSS ≥ 16, *n* = 19), respectively. Blood samples were collected immediately after hospitalization, followed by transthoracic echocardiographic examination. The results showed that the flow cytometric analysis for assessment of inflammatory biomarkers of TLR2+/CD14+cells, TLR4+/CD14+cells, Ly6g+/CD14+cells, and MPO+/CD14+cells, and ELISA assessment for circulatory level of sST2 were significantly higher in groups 2/3 than in group 1 (all *p* < 0.01). However, these parameters did not show significant differences between groups 2 and 3 (all *p* > 0.05). The LVEF was significantly lower in group 3 than in group 1 (*p* < 0.001), but it displayed no difference between groups 1/2 or between groups 2/3. These inflammatory biomarkers ((TLR2+/CD14+cells// TLR4+/CD14+cells// MPO+/CD14+cells) and sST2)) were significantly positively correlated to NIHSS and strongly negatively correlated to LVEF (all *p* < 0.05). Multivariate analysis demonstrated that both MPO/CD14+cells >20% (*p* = 0.027) and sST2 ≥ 17,600 (*p* = 0.004) were significantly and independently predictive of moderate-severe CCS after acute IS. Receiver operating characteristic curve analysis demonstrated that sST2 was the most powerful predictor of CCS with a sensitivity of 0.929 and a specificity of 0.731 (*p* < 0.001). In conclusion, sST2 is a useful biomarker for prediction of CCS severity in patients after acute IS.

## 1. Introduction

Ischemic stroke (IS), a growing epidemic issue, is the leading cause of long-term disability and the second cause of death worldwide [1,2,3,4]. Thus, it is undoubtedly an extremely important issue and always raises the investigators’ interest to find out independently prognostic predictors for patients suffering from acute IS [5,6,7,8]. These predictors are required not only to be simple and unique but also have high sensitivity and specificity [5,6,7,8]. Of these prognostic predictive parameters, circulatory inflammatory biomarkers have been broadly identified and extensively investigated. In fact, plentiful studies have previously demonstrated that circulating levels of inflammatory biomarkers are substantially increased in patients after acute IS [6,9,10,11,12]. Intriguingly, these circulating inflammatory biomarkers were identified as much more increased in severe IS patients than in those with a mild IS [6,9,10,11,12]. Furthermore, a strong correlation between severity of brain damage and systemic inflammatory reactions has also been established [6,9,10,11,12,13,14,15,16]. 

The relationship between cardiac and neurological functions is closely linked. For example, optimal control of blood pressure is utmost crucial for improvement of clinical outcome among patients after acute IS. This important brain–heart interaction has been further proven by our recent study [16] which has identified that acute IS patients with higher neurological dysfunction had lower left ventricular ejection fractions (LVEF) and higher neutrophil-to-lymphocyte ratios and platelet-to-lymphocyte ratios compared to those with lower National Institute of Health Stroke Scale (NIHSS) ratings. Undoubtedly, cardiovascular disease and cerebrovascular disease share a majority of similar atherosclerotic risk factors, such as hypertension, diabetes mellitus, and hyperlipidemia. Thus, coexistence of cardiovascular disease and cerebrovascular disease is like “two sides of the same coin”, i.e., so-called “cerebral–cardiac syndrome (CCS)”.

Interleukin (IL)-33, a new member of the IL-1 family of cytokines, promotes Th2 type immune responses by signaling through the suppression of tumorigenesis 2 ligand (ST2L) and IL-1RAcP dimeric receptor complex [17,18]. Additionally, the biological effects of IL-33 are limited by a soluble decoy form of ST2 (i.e., sST2). Abundant data have shown that IL-33/ST2 pathway plays an essential role of chronic inflammatory cardiovascular diseases, atherosclerosis, obesity, cardiac remodeling, and myocardial fibrosis, as well as acts as an independent predictor of mortality in patients with heart failure or myocardial infarction [17,18,19,20,21,22]. However, whether the sST2 can be applied as a novel biomarker for additive risk stratification in patients with acute IS, or even CCS, is currently unclear.

## 2. Materials and Methods

### 2.1. Study Design

The study design has been clearly described in our recent report [16]. In detail, this was a prospective clinical study performed in a tertiary medical center of southern Taiwan between November 2015 and October 2017. The study protocol was approved by the institutional review boards (IRB number: 104-5222B) of Kaohsiung Chang Gung Memorial Hospital and written informed consent was obtained from all participants prior to enrollment.

### 2.2. Inclusion and Exclusion Criteria

The inclusion and exclusion criteria have been reported in our recent study [15,16]. In detail, eligible patients aged between 45 and 80 years with acute IS regardless of thrombolytic or endovascular therapy were prospectively enrolled into the present study. Acute IS was diagnosed by neurologists based upon detailed clinical assessment, neurological examination, and image modalities which included brain computed tomography or brain magnet resonance imaging. 

The exclusion criteria comprised transient ischemic attack, young stroke, cerebellar infarcts, acute hemorrhagic stroke, traumatic brain injury, active infection without treatment, autoimmune diseases, malignancy with life expectancy less than one year, myocardial infarction less than one month, major surgery within three months, advanced liver cirrhosis, and end-stage renal disease on peritoneal dialysis or hemodialysis. Additionally, patients presenting with hemodynamic instability, post cardiopulmonary resuscitation, or indication for immediate surgical intervention were also excluded from the present study. 

### 2.3. Categorization of Stroke into Mild, Moderate, and Severe Neurological Dysfunctions

The quantification of stroke severity was performed by using National Institute of Health Stroke Scale (NIHSS) [23]. In addition, we evaluated the scales of neurologic deficit with NIHSS (0–42) within 12 h of stroke and global disability severity with the modified Rankin stroke scale (MRS) (0–6). On the basis of the previous reports [24,25], the patients with an NIHSS ≤8 are highly likely to have good clinical outcomes, whereas those with higher NIHSS expressed more severe stroke with poorer prognostic outcomes. Therefore, we categorized the patients into mild IS (i.e., NIHSS ≤8), moderate IS (i.e., NIHSC=9–15), and severe IS (i.e., NIHSS ≥ 16), respectively.

### 2.4. Patients’ Enrollment

The detailed information regarding the sample size calculation has been reported in our recent study [16]. Between November 2015 and October 2017, a total of 110 consecutive subjects who met the inclusion and exclusion criteria were prospectively enrolled into the study. We further excluded 11 cases with hemorrhagic transformation (*n* = 4), life-threatening stress ulcer bleeding (*n* = 3), concomitant heart attack (*n* = 1), complications of aortic dissection (*n* = 1), and another hospital transfer (*n* = 2) after enrollment. Finally, 99 patients were enrolled into the study. All patients were completely surveyed during hospitalization and objectively assessed for in-hospital laboratory and clinical outcomes.

### 2.5. Flow Cytometric Analysis for Assessment of Circulatory Cells

Flow cytometric analyses of circulating levels of toll-like receptor (TLR)2+/CD14+ cells, TLR4+/CD14+ cells, Ly6g+/CD14+ cells, and myeloperoxidase (MPO)+/CD14+ cells, four indices of inflammation, were performed by a senior technician who has expertise in flow cytometric analysis and is blinded to the study design, grouping, and treatment strategies. The fluorescence-activated cell sorter machine (FACSCaliburTM system; Beckman Coulter Inc, Brea, CA, USA) was utilized for flow cytometric analysis in the present study.

### 2.6. ELISA Assessment for Circulating Levels of Proinflammatory Cytokines on Admission

Circulating levels of interleukin (IL)-33 and sST2, two soluble proinflammatory cytokines, were measured by duplicated determination with a commercially available ELISA method (R&D Systems, Minneapolis, MN, USA). Intra-observer variability of the measurements was also assessed and the mean intra-assay coefficients of variance were all <4.5%.

### 2.7. Medications for the Study Patients

Aspirin was prescribed for all acute IS patients unless contraindicated. Clopidogrel was prescribed if the patient did not tolerate or was allergized to aspirin. As for those with atrial fibrillation (AF)-related cardioembolic, warfarin or direct oral anticoagulant was prescribed after neurological condition became stable [26]. Other comorbidities or underlying diseases were treated with guideline-direct medications, including statins, oral antidiabetic agents, renin-aldosterone system (RAS) inhibitors, diuretics, calcium channel blockers, and beta blockades.

### 2.8. Echocardiographic Measurement for LV Systolic Function and Grade of Valvular Regurgitation

All IS subjects in neurology wards or intensive care units received echocardiographic study within 5 days after stroke. Echocardiographic study was performed by a cardiologist who was blinded to the severity of stroke and study allocation. To evaluate cardiac chamber size, LVEF, and grade of mitral regurgitation (MR), conventional echocardiography was performed with standard 2-dimenional (2D) views, M-mode, tissue, and color Doppler assessment. Digital images were collected and data were analyzed according to the standardized echo protocol [27]. Cardioprotective drugs were also adjusted in time according to abnormal findings.

### 2.9. Definition of Severity of CCS

After echocardiographic assessment, the severity of CCS was further classified into mild and moderate-severe CCS according to NIHSS score and LVEF. Mild CCS was defined as NIHSS ≤8 and LVEF ≥60%, i.e., mild damage of brain and deterioration of heart function. On the other hand, moderate-severe CCS was defined as NIHSS >8 and LVEF <60%, i.e., more predominant brain injury and cardiac dysfunction.

#### Statistical Analysis

Independent t and Mann–Whitney U tests were used to compare the difference between groups for continuous variables as appropriate. For discrete or categorical variables, chi-square and Fisher exact tests were applied to detect the proportions between groups. Additionally, Pearson’s or Spearman’s correlation analysis was adopted to assess the relationship of NIHSS to LVEF. Area under the curve (AUC) of receiver operating characteristic (ROC) curve and Youden’s index were further used for calculating cutoff value of mild or moderate-severe CCS. Finally, we performed logistic regression model with univariate and multivariate analyses to identify potential independent predictors of mild or moderate-severe CCS. Statistical analysis was performed using SPSS statistical software for Windows version 22 (SPSS for Windows, version 22; SPSS, IL, USA). A value of *p* < 0.05 was considered statistically significant.

## 3. Results

### 3.1. The Baseline Characteristics of IS Patients in Three Groups (Table 1)

Table 1 shows upon admission, the average NIHSS was significantly higher in moderate (group 2) and severe IS (group 3) patients than in mild IS (group 1) counterparts, but it showed no difference between groups 2 and 3. There were no significant differences in terms of age, sex, current smoking, diabetes mellitus, hypertension systolic and diastolic blood pressure, and rates of old myocardial infarction, old stroke, atrial fibrillation, stain, or RAS inhibitor use. Additionally, the white blood cell count, platelet count, and hemoglobin did not differ among the three groups. However, the circulating levels of segment and lymphocyte were significantly lower in group 1 than in groups 2 and 3, but they exhibited no difference between the latter two groups.

The circulating levels of creatinine, total cholesterol, high-density lipoprotein, and low-density lipoprotein cholesterol were similar among the three groups. The triglyceride level was higher in group 1 than in groups 2 and 3, but no difference between groups 2 and 3.

Flow cytometric analysis demonstrated that the circulating level of TLR2+/CD14+ cells was significantly higher in group 3 than in groups 1 and 2, but no difference between groups 1 and 2. On the other hand, the circulating level of TLR4+/CD14+ cells was significantly lower in group 1 than in group 3, but similar between groups 1 and 2 or between groups 2 and 3. The flow cytometric analysis further showed that the circulating level of MPO+/CD14+ cells was significantly higher in group 3 than in groups 1 and 2, and significantly higher in group 2 than in group 1. However, the circulating number of Ly6g+/CD14+ cells did not differ among the three groups.

The ELISA result demonstrated that the circulating level of sST2 was more significantly increased in groups 2 and 3 than in group 1, but no difference between groups 2 and 3. By contrast, the circulating level of IL-33 did not differ among groups. As for echocardiographic results, the LVEF was significantly higher in group 1 than group 3, but it did not differ between groups 1 and 3 or between groups 2 and 3. On the other hand, the mean degree of MR and the in-hospital mortality rate were similar among the three groups.

### 3.2. Correlation of Circulatory Inflammatory Biomarkers to the Severity of Neurological Dysfunction and Impairment of Heart Function (Table 2)

As shown in Table 2, to elucidate the correlation of circulatory inflammatory biomarkers to the severity of neurological dysfunction (NIHSS) and to the impairment of heart function (LVEF), the flow cytometric analysis and ELISA method were utilized in the present study. The result demonstrated that the circulating levels of TLR2+/CD14+ cells, TLR4+/CD14+ cells, and MPO+/CD14+ cells via flow cytometric assessment and sST2 level via ELISA assessment were significantly positively correlated to the severity of IS and significantly negatively correlated to cardiac dysfunction. On the other hand, there was no significant relationship of the two inflammatory biomarkers (i.e., Ly6G+/CD14+ cells, IL33) to NIHSS score or LVEF level.

### 3.3. ROC Curve and Youden’s Index for Determining Cutoff Value of the Parameters According to NIHSS, LVEF, and both (Table 3)

Table 3A shows downhill change of LVEF, percentage of MPO+/CD14+ cells, and value of sST2 were highly correlated with NIHSS >8 (AUC >0.7 and *p* < 0.001). Similarly, as shown in Table 3B, LVEF <60% was found closely linked to the changes of the NIHSS and the levels of MPO+/CD14+ cells and sST2. Furthermore, Table 3C demonstrates the higher correlation was found between mild degree of CCS (i.e., NIHSS ≤8 or LVEF ≥60%) and the levels of MPO+/CD14+ cells and sST-2. The above similar trend was also observed in the moderate-severe degree of CCS (Table 3D), suggesting the levels of MPO+/CD14+ cells and sST-2 had the potential to determine the severity of CCS. Of these two parameters, sST2 was identified to have greater discriminating ability for CCS severity, and its cutoff value for the mild and moderate-severe CCS was 13,830 and 17,643, respectively.

### 3.4. Predictors of the Mild CCS (Table 4) and Moderate-Severe CCS (Table 5)

The Table 4 demonstrates logistic regression analysis was performed to identify independent predictors of mildest CCS (NIHSS ≤8 and LVEF ≥60%). The results demonstrated that age, dyslipidemia, atrial fibrillation, triglyceride, MR (grades 2 to 4), mean TLR2+/CD14+ cells, TLR2+/CD14+ cells <25%, TLR4+/CD14+ cells <0.25%, mean MPO+/CD14+cells, MPO+CD14+ cells <20%, sST2 <14,000 (pg/mL), and mean sST2 were significantly predictive of mild CCS. After multivariate analysis, we found that sST2 <14,000 (pg/mL) was the only independent predictor of the mildest CCS. 

Table 5 lists the results of independent factors predictive of the moderate to severe CCS (NIHSS >8 and LVEF <60%) using univariate and multivariate analyses. Age, moderate to severe MR, mean TLR2+/CD14+cells (%), TLR2+CD14+ ≥25%, TLR4+/CD14+cells ≥0.25%, mean Ly6G+/CD14+cells (%), MPO+/CD14+cells (%), MPO+/CD14+cells ≥20%, and mean sST-2 and sST2 ≥17,600 (pg/mL) were potentially predictive of moderate-severe CCS. Further multivariate analysis demonstrated that only MPO+/CD14+cells ≥20% and sST2 ≥17,600 (pg/mL) were the independent predictors of moderate-severe CCS. Taken together, the values of sST2 <14,000 and ≥17,600 (pg/mL) were importantly recognized as the strongest predictors of the mild and moderate-severe CCS, respectively, with a very significant odd ratio and discriminating value.

## 4. Discussion

The present study designed to investigate whether circulating inflammatory biomarkers act as simple and useful predictors of CCS severity in patients after acute IS reveals several striking clinical information. First, increases in circulatory inflammatory cells (i.e., TLR2+/CD14+, TLR4+/CD14+, MPO+/CD14+) and proinflammatory cytokine (i.e., sST2) were found to be predictive of concomitant neurological and cardiac systolic dysfunction. Second, among all of the inflammation-relevant variables, the percentage of MPO+/CD14+cells (with a cutoff value of ≥20%) and the level of sST2 (with the cutoff value of ≥17,600 (pg/mL)) were the only two independent biomarkers predictive of moderate-severe CCS. Third, of these two biomarkers, sST2 was most strongly predictive of severity of CCS, highlighting that the sST2 with a cutoff value of ≥17,600 (pg/mL) was an utmost important biomarker in our clinical practice to categorize the risk stratification in patients with acute IS.

One important finding in the present study was that as compared with the mild IS patients, the circulating levels of inflammatory cells and proinflammatory cytokines were remarkably increased in severe IS patients. Interestingly, previous studies have established that circulating levels of inflammatory biomarkers are significantly increased in patients after acute IS [6,9,10,11,12]. Of particular concern was that these circulating inflammatory biomarkers were recognized to be much more upregulated in severe IS patients than in those of patients with a mild IS [6,9,10,11,12]. Our finding was, therefore, consistent with those in previous studies [6,9,10,11,12]. 

Previous study has emphasized that the cardiovascular disease and cerebrovascular disease are “two sides of the same coin”, with not only sharing common atherosclerotic risk factors but also being mediated by damage-associated molecular patterns [7]. In the present study, when we took a look at the cerebral–cardiac axis in patients after acute IS, the LVEF, an index of LV function partially affected by moderate to severe MR, was markedly more reduced in severe IS patients than in mild IS patients. Additionally, there was a strong correlation between increased circulatory inflammatory biomarkers and impaired LVEF (refer to Table 2). Intriguingly, a link between an increase in inflammatory biomarkers and LV dysfunction/heart failure has been clearly elucidated in settings of IS or brain damage with any etiology [28,29,30,31]. Again, our finding was comparable with the findings of previous studies [7,28,29,30,31] regarding the complex brain–heart interaction.

Undoubtedly, mild grade of IS commonly has the best prognostic outcome among all IS patients. In the present study, we categorized the group of patients with mild CCS (i.e., with NIHSS <8 and LVEF >60%) as the mild IS group concomitant with or followed by less cardiac dysfunction. Interestingly, the circulating level of sST2 <14,000 (pg/mL) was independently predictive of mild CCS, suggesting that circulating sST2 less than 14,000 (pg/mL) checked at the moment of acute IS would be a good prognostic biomarker for the IS or CCS victims. 

Previous studies have clearly revealed that the sST2 pathway plays a crucial role in inflammatory cardiovascular diseases, atherosclerosis, and myocardial fibrosis, and is independently predictive of mortality in patients with heart failure or myocardial infarction [17,18,19,20,21,22]. However, there is still lacking data regarding the relation between circulating level of sST2 and the prognostic outcome in patients after acute IS, especially for CCS. In the present study, after multivariate adjustment, we found only two parameters (i.e., MPO+/CD14+cells ≥20%, sST2 ≥17,600 [pg/mL]) were delineated as the significantly independent predictors of moderate-severe CCS and only one parameter of sST2 <14,000 (pg/mL) to be associated with mild CCS, indicating checking sST2 not only predicted the mildest CCS but also the most severe CCS. Therefore, our study established the role of sST2 on discriminating severity of CCS following acute IS.

This study has limitations. First, the sample size was relatively small, especially after allocation into specific groups. Accordingly, some analytical significance would be distorted in the present study. Second, due to an extremely low incidence rate of clinical events, the association between value of sST2 and clinical outcome of CCS, e.g., mortality, was regrettably unable to be analyzed. Third, the study period was also relatively short (i.e., only during hospitalization), and the correlation of increased circulating levels of biomarkers to long-term prognostic outcome was beyond the scope of the present study. Fourth, this study did not perform the correlation between the circulating levels of inflammatory biomarkers and the duration of hospitalization. Thus, we did not provide information regarding who would be hospitalized for longer than the others. Finally, because the treatment was based on guidelines and similar among the IS patients, this study did not provide information with regard to the impact of different treatment strategies on circulatory levels of inflammatory biomarkers and clinical outcome in patients after acute IS.

## 5. Conclusions

In conclusion, the results of the present study demonstrated that sST2 was a superb biomarker for prediction of CCS severity in patients after acute IS.

## Figures and Tables

**Table 1:** Baseline characteristics of three groups with different severities of acute ischemic stroke (IS).

Variables	Mild IS (*n* = 66) *	Moderate IS (*n* = 14) *	Severe IS (*n* = 19) *	*p*-Value
Average NIHSS	3.64 ± 2.03 ^a^	12.21 ± 2.33 ^b^	23.11 ± 6.86 ^b^	<0.001
Age, year	62.44 ± 12.08	65.93 ± 8.86	69.89 ± 10.85	0.094
Sex (male), *n* (%)	36 (54.5%)	9 (64.3%)	14 (73.7%)	0.302
Smoker, *n* (%)	25 (37.9%)	5 (35.7%)	2 (10.5%)	0.077
Hypertension, *n* (%)	51 (77.3%)	11 (78.6%)	17 (89.5%)	0.376
Diabetes mellitus, *n* (%)	23 (34.8%)	7 (50.0%)	4 (21.1%)	0.221
Dyslipidemia, *n* (%)	35 (53.0%)	2 (14.3%)	8 (42.1%)	0.029
Old MI, *n* (%)	2 (3.0%)	0 (0.0%)	1 (5.3%)	1.000
SBP, mmHg	165.97 ± 30.35	153.07 ± 28.38	154.37 ± 23.25	0.147
DBP, mmHg	91.21 ± 17.40	83.86 ± 18.75	85.05 ± 14.81	0.236
Old stroke, *n* (%)	11 (16.7%)	3 (21.4%)	5 (26.3%)	0.367
Atrial fibrillation, *n* (%)	6 (9.1%)	0 (0.0%)	5 (26.3%)	0.499
ACEI or ARB, *n* (%)	35 (53.0%)	7 (50.0%)	5 (26.3%)	0.119
Statin, *n* (%)	38 (57.6%)	4 (28.6%)	11 (57.9%)	0.130
WBC count, 1000/μL	8.18 ± 2.65	9.11 ± 2.64	8.56 ± 3.01	0.484
Segment, %	63.52 ± 12.35 ^a^	74.55 ± 9.52 ^b^	70.83 ± 14.69 ^b^	0.004
Lymphocyte, %	28.39 ± 11.38 ^a^	18.75 ± 7.92 ^b^	18.50 ± 11.57 ^b^	<0.001
Hemoglobin, g/dL	14.22 ± 1.79	13.91 ± 2.11	14.33 ± 2.80	0.840
Platelet count, 1000/μL	214.70 ± 76.24	222.36 ± 44.26	195.74 ± 53.32	0.480
Creatinine, mg/dL	1.13 ± 0.85	1.24 ± 1.41	1.37 ± 0.95	0.095
Total cholesterol, mg/dL	183.42 ± 49.45	193.36 ± 61.61	186.00 ± 37.22	0.777
HDL, mg/dL	43.29 ± 14.30	40.29 ± 10.22	47.94 ± 8.56	0.066
LDL, mg/dL	100.55 ± 45.79	118.21 ± 59.65	109.00 ± 37.90	0.400
Triglyceride, mg/dL	141.02 ± 74.35 ^a^	117.50 ± 52.76 ^a, b^	95.17 ± 47.05 ^b^	0.041
TLR2+CD14+, %	21.14 ± 8.20 ^a^	25.04 ± 8.73 ^a^	36.91 ± 14.30 ^b^	<0.001
TLR4+CD14+, %	0.62 ± 2.94 ^a^	0.50 ± 0.51 ^a, b^	0.47 ± 0.53 ^b^	0.003
Ly6g+CD14+, %	4.96 ± 5.23	5.99 ± 6.05	7.00 ± 9.24	0.968
MPO+CD14+, %	14.99 ± 11.04 ^a^	23.20 ± 12.85 ^b^	32.67 ± 16.86 ^c^	<0.001
Interleukin 33 (pg/mL)	1.92 ± 1.49	1.46 ± 0.71	3.02 ± 5.21	0.396
ST2 (pg/mL)	15855 ± 13056 ^a^	23139 ± 15194 ^b^	35459 ± 21030 ^b^	<0.001
2-D echocardiography				
LVEF, %	67.72 ± 9.40 ^a^	62.55 ± 11.04 ^a,b^	56.59 ± 11.99 ^b^	<0.001
MR (2-4), n (%)	15 (24.2%)	3 (23.1%)	8 (47.1%)	0.213
Mortality, n (%)	0 (0.0%)	0 (0.0%)	2 (10.5%)	0.109

Data are expressed as means ± standard deviation, or *n* (%). Abbreviation: NIHSS, National Institute of Health Stroke Scale; IS, ischemic stroke; SBP, systolic blood pressure; DBP, diastolic blood pressure, MI, myocardial infarction; ACEI, angiotensin-converting-enzyme inhibitor; ARB, angiotensin II receptor blocker; WBC, white blood cell; HDL, high-density lipoprotein; LDL, low-density lipoprotein; TLR, toll-like receptor; MPO, myeloperoxidase; LVEF, left ventricular ejection fraction; MR, mitral regurgitation. * Mild IS (group 1), moderate IS (group 2), and severe IS (group 3) was defined as NIHSS ≤8, 9–15, and ≥16, respectively. Letters (a, b, c) indicate significance (at 0.05 level) (by Scheffé’s multiple comparison analysis).

**Table 2:** Correlation of circulatory inflammatory biomarkers to the severity neurological dysfunction and impairment of heart function.

Variables	Correlation Coefficient (R)	*p*-Value
Severity of stroke		
NIHSS vs. TLR2+/CD14+ cells	0.392	<0.001
NIHSS vs. TLR4+/CD14+ cells	0.237	0.018
NIHSS vs. Ly6g+/CD14+ cells	0.009	0.930
NIHSS vs. MPO+/CD14+ cells	0.305	0.003
NIHSS vs. IL33	0.075	0.463
NIHSS vs. sST2	0.511	<0.001
Degree of LV dysfunction		
LVEF vs. TLR2+/CD14+ cells	−0.228	0.028
LVEF vs. TLR4+/CD14+ cells	−0.231	0.025
LVEF vs. Ly6g+/CD14+ cells	−0.083	0.425
LVEF vs. MPO+/CD14+ cells	−0.219	0.037
LVEF vs. IL33	0.026	0.800
LVEF vs. sST2	−0.272₋	0.008

Abbreviation: TLR, toll-like receptor; IL, interleukin; LVEF, left ventricular ejection fraction; NIHSS, National Institute of Health Stroke Scale; R, Pearson’s or Spearman’s correlation coefficient; vs., versus.

**Table 3:** Area under the curve (AUC) and Youden’s index for determining cutoff value of variables based on NIHSS and LVEF.

Variable	AUC (*p*-Value)	Youden’s Index	Cutoff Value	Sensitivity	Specificity
NIHSS >8 (**A**)					
LVEF (%)	0.735 (<0.001)	0.450	34.45	0.741	0.710
TLR2+/CD14+cells (%)	0.695 (0.004)	0.367	25.5	0.593	0.774
TLR4+/CD14+cells (%)	0.667 (0.013)	0.312	0.17	0.667	0.645
Ly6g+CD14+cells (%)	0.513 (0.851)	0.164	4.15	0.519	0.645
MPO+CD14+cells (%)	0.735 (<0.001)	0.370	19.15	0.741	0.629
IL-33 (pg/mL)	0.467 (0.627)	0.071	0.5	0.926	0.145
sST2 (pg/mL)	0.780 (<0.001)	0.539	14118	0.926	0.613
LVEF <60% (**B**)					
NIHSS	0.730 (0.001)	0.487	12	0.609	0.879
MRS	0.680 (0.011)	0.321	4	0.609	0.712
TLR2+/CD14+cells (%)	0.654 (0.028)	0.336	25.1	0.609	0.727
TLR4+/CD14+cells (%)	0.667 (0.018)	0.332	0.17	0.696	0.636
Ly6g+CD14+cells (%)	0.615 (0.101)	0.289	14.1	0.304	0.985
MPO+CD14+cells (%)	0.733 (0.001)	0.432	17.4	0.826	0.606
IL-33 (pg/mL)	0.430 (0.320)	0.045	0.33	1.000	0.045
sST2 (pg/mL)	0.734 (0.001)	0.474	13,830	0.913	0.561
NIHSS >8 and LVEF <60% (**C**)					
TLR2+/CD14+cells (%)	0.739 (0.005)	0.458	25.5	0.714	0.744
TLR4+/CD14+cells (%)	0.747 (0.003)	0.498	0.17	0.857	0.641
Ly6g+CD14+cells (%)	0.556 (0.507)	0.247	16.25	0.286	0.962
MPO+CD14+cells (%)	0.802 (<0.001)	0.557	19.55	0.929	0.628
IL-33 (pg/mL)	0.469 (0.712)	0.095	0.7	0.929	0.167
sST2 (pg/mL)	0.806 (<0.001)	0.659	17,643	0.929	0.731
NIHSS ≤8 and LVEF ≥60% (**D**)					
MRS	0.819 (<0.001)	0.590	4	0.738	0.852
TLR2+/CD14+cells (%)	0.706 (0.001)	0.393	25	0.619	0.774
TLR4+/CD14+cells (%)	0.670 (0.004)	0.315	0.17	0.667	0.648
Ly6g+CD14+cells (%)	0.564 (0.284)	0.214	4	0.548	0.667
MPO+CD14+cells (%)	0.748 (<0.001)	0.385	17.5	0.737	0.648
IL-33 (pg/mL)	0.460 (0.504)	0.056	0.35	1.000	0.056
sST2 (pg/mL)	0.788 (<0.001)	0.553	13,830	0.905	0.648

Abbreviation: AUC = area under the receiver operating characteristic curve; NIHSS = National Institute of Health Stroke Scale; MRS = modified Rankins scale; LVEF = left ventricular ejection fraction; NLR = neutrophil-to-lymphocyte ratio; PLR, platelet-to-lymphocyte ratio; TLR = toll-like receptor; CD = cluster of differentiation; Ly6G = lymphocyte antigen 6 complex locus G6D; MPO = myeloperoxidase; IL33 conc. = interleukin 33 concentration; ST2 = suppression of tumorigenesis 2; ST2-IL33R conc. = antibody concentration of ST2-IL33 receptor.

**Table 4:** Predictors of the mild CCS (NIHSS ≤8 and LVEF ≥60%).

Severity of CCS	Univariate Analysis			Multivariate Analysis		
Variables	OR	95% CI	*p*-Value	OR	95% CI	*p*-Value
Age per year	0.955	0.919–0.992	0.018			
Age ≥65 years	0.386	0.166–0.900	0.027			
Male sex	0.895	0.392–2.045	0.793			
Smoker	2.200	0.900–5.378	0.084			
Systolic blood pressure	1.012	0.998–1.027	0.100			
Diastolic blood pressure	1.013	0.989–1.038	0.279			
Hypertension	0.476	0.165–1.370	0.169			
Diabetes mellitus	0.758	0.321–1.791	0.528			
Dyslipidemia	2.588	1.112–6.024	0.027			
Old myocardial infarction	1.577	0.138–18.004	0.714			
Old stroke	0.420	0.147–1.200	0.105			
Atrial fibrillation	0.141	0.029–0.694	0.016			
RAS inhibitor	1.471	0.651–3.322	0.354			
Statin	1.375	0.612–3.088	0.441			
Leukocyte	0.945	0.815–1.096	0.457			
Hemoglobin	1.136	0.928–1.390	0.218			
Platelet	1.002	0.996–1.008	0.563			
Creatinine	0.903	0.595–1.372	0.633			
Total cholesterol	1.002	0.994–1.010	0.633			
High-density lipoprotein	1.001	0.971–1.033	0.933			
Low-density lipoprotein	0.998	0.989–1.007	0.655			
Triglyceride	1.010	1.003–1.018	0.008			
Mitral regurgitation (grade 2–4)	0.334	0.131–0.854	0.022			
TLR2+/CD14+cells (%)	0.928	0.887–0.971	0.001			
TLR2+/CD14+cells <25%	5.000	2.068–12.089	<0.001			
TLR4+/CD14+cells (%)	1.048	0.857–1.281	0.650			
TLR4+/CD14+cells <0.25%	3.143	1.332–7.416	0.009			
Ly6G+/CD14+cells (%)	0.937	0.875–1.004	0.066			
MPO+/CD14+cells (%)	0.926	0.889–0.964	<0.001			
MPO+/CD14+cells <20%	3.846	1.600–9.246	0.003			
IL33 (pg/mL)	0.980	0.841–1.143	0.801			
sST2 (pg/mL)	1.000	1.000–1.000	0.002			
sST2 <14,000 (pg/mL)	13.632	4.592–40.469	<0.001	12.743	3.836–42.328	<0.001

Abbreviation: CCS = cerebral-cardiac syndrome; NIHSS = National Institute of Health Stroke Scale; LVEF = left ventricular ejection fraction; OR = odds ratio; CI = confidence interval; RAS = renin-angiotensin-system; TLR = toll-like receptor; CD = cluster of differentiation; Ly6G = lymphocyte antigen 6 complex locus G6D; MPO = myeloperoxidase; IL33 = interleukin 33; sST2 = soluble suppression of tumorigenesis 2.

**Table 5:** Predictors of the moderate to severe CCS (NIHSS >8 and LVEF <60%).

Severity of CCS	Univariate Analysis			Multivariate Analysis		
Variables	OR	95% CI	*p*-Value	OR	95% CI	*p*-Value
Age per year	1.061	1.004–1.121	0.037			
Age ≥65 years	2.854	0.848–9.604	0.090			
Male sex	2.217	0.658–7.476	0.199			
Smoker	0.619	0.183–2.100	0.442			
Systolic blood pressure	0.998	0.980–1.017	0.850			
Diastolic blood pressure	0.985	0.954–1.018	0.364			
hypertension	0.750	0.213–2.637	0.654			
Diabetes mellitus	0.556	0.164–1.879	0.345			
Dyslipidemia	0.631	0.209–1.901	0.413			
Old myocardial infarction	2.600	0.221–30.531	0.447			
Old stroke	0.230	0.028–1.859	0.168			
Atrial fibrillation	3.476	0.882–13.705	0.075			
RAS inhibitor	0.302	0.090–1.015	0.053			
Statin	1.052	0.357–3.102	0.927			
Leukocyte	0.964	0.782–1.189	0.733			
Hemoglobin	1.038	0.992–1.085	0.108			
Platelet	0.995	0.987–1.003	0.240			
Creatinine	1.474	0.944–2.302	0.088			
Total-cholesterol	1.003	0.992–1.014	0.587			
High-density lipoprotein	1.018	0.980–1.059	0.353			
Low-density lipoprotein	1.001	0.989–1.012	0.921			
Triglyceride	0.991	0.981–1.002	0.097			
Mitral regurgitation (grade 2-4)	3.222	1.058–9.812	0.039			
TLR2+/CD14+cells (%)	1.093	1.036–1.153	0.001			
TLR2+CD14+ ≥25%	6.875	2.012–23.497	0.002			
TLR4+/CD14+cells (%)	1.003	0.809–1.245	0.977			
TLR4+/CD14+cells ≥0.25%	7.909	2.304–27.152	0.001			
Ly6G+/CD14+cells (%)	1.091	1.012–1.177	0.024			
MPO+/CD14+cells (%)	1.088	1.035–1.143	0.001			
MPO+/CD14+cells ≥20%	10.345	2.162–49.490	0.003	6.633	1.244–35.376	0.027
IL-33 (pg/mL)	1.127	0.944–1.346	0.186			
sST2 (pg/mL)	1.000	1.000–1.000	0.021			
sST2 ≥17,600 (pg/mL)	37.174	4.638–297.96	0.001	23.448	2.794–196.801	0.004

Abbreviation: CCS = cerebral-cardiac syndrome; NIHSS = National Institute of Health Stroke Scale; LVEF = left ventricular ejection fraction; OR = odds ratio; CI = confidence interval; RAS = renin-angiotensin-system; TLR = toll-like receptor; CD = cluster of differentiation; Ly6G = lymphocyte antigen 6 complex locus G6D; MPO = myeloperoxidase; IL-33 = interleukin 33; sST2 = soluble suppression of tumorigenesis 2.

## Abbreviations

**IS**	**Ischemic Stroke**
LVEF	left ventricular ejection fraction
NIHSS	National Institute of Health Stroke Scale
CCS	Cerebral–cardiac syndrome
IL-33	interleukin-33
ST2L	suppression of tumorigenesis 2 ligand
MRS	modified Rankin stroke scale
TLR	toll-like receptor
MPO	myeloperoxidase
AF	atrial fibrillation
RAS	renin-aldosterone system
MR	mitral regurgitation
AUC	area under the curve
ROC	receiver operating characteristic
